# Geographical Distribution of Methanogenic Archaea in Nine Representative Paddy Soils in China

**DOI:** 10.3389/fmicb.2016.01447

**Published:** 2016-09-13

**Authors:** Qianhui Zu, Linghao Zhong, Ye Deng, Yu Shi, Baozhan Wang, Zhongjun Jia, Xiangui Lin, Youzhi Feng

**Affiliations:** ^1^State Key Laboratory of Soil and Sustainable Agriculture, Institute of Soil Science, Chinese Academy of Sciences, NanjingChina; ^2^Department of Chemistry, Pennsylvania State University, Mont Alto, PAUSA; ^3^CAS Key Laboratory of Environmental Biotechnology, Research Center for Eco-Environmental Sciences, Chinese Academy of Sciences, BeijingChina

**Keywords:** paddy soil, methanogenic archaea, geographical distribution, latitude, soil properties

## Abstract

Paddy field methanogenic archaea are responsible for methane (CH_4_) production and contribute significantly to climate change. The information regarding the spatial variations in the abundance, the diversity and the composition of such ecologically important microbes, however, is quite limited at large scale. In this investigation, we studied the abundance, alpha diversity and geographical distribution of methanogenic archaeal communities in nine representative paddy sites, along a large latitudinal gradient in China, using pyrosequencing and real-time quantitative PCR. It is found that all paddy soils harbor constant methanogenic archaeal constituents, which is dominated by family Methanocellaceae (37.3%), Methanobacteriaceae (22.1%), Methanosaetaceae (17.2%), and Methanosarcinaceae (9.8%). Methanogenic archaeal abundance is primarily influenced by soil C (*R* = 0.612, *P* = 0.001) and N (*R* = 0.673, *P* = 0.001) contents, as well as alpha diversity by soil pH (PD: *R* = -0.552, *P* = 0.006; Chao1: *R* = -0.615, *P* = 0.002). Further exploration revealed that both spatial distance (*R* = 0.3469, *P* = 0.001, partial mental test) and soil chemical variables mainly about soil C and N (*R* = 0.2847, *P* = 0.001) are the two major factors affecting methanogenic archaeal community composition distribution in paddy soils. This finding will allow us to develop a better picture of the biogeographic ranges of these ecologically important microbes and get deeper insights into their ecology.

## Introduction

Rice is the world’s most important agronomic plant, with nearly 150 million ha under cultivation globally (Roger, 1996). More than half of the world’s rice grows in paddy fields under flooded (or anoxic) conditions. As a result, paddy fields are one of major anthropogenic sources for atmospheric methane (CH_4_). IPCC (2013) reported 33–40 Tg CH_4_ year^-1^ emission from paddy fields, equivalent to 12.5% of anthropogenic CH_4_, or 5.0% of annual global CH_4_ emission. Methanogenesis is the final degradation process of organic matter in paddy fields. During this process, organic matters with large molecular weight in paddy soil are anoxically degraded into small molecules, such as methanol, acetate, formate and H_2_/CO_2_, by hydrolytic and fermenting microbes. Bacterial reducers of nitrate, Fe(III) and sulfate in paddy soil are thermodynamically more competitive with electron donors than methanogenic archaea, and they outcompete methanogens for utilizing these small molecular weight organic carbon and/or H_2_/CO_2_ to conduct biogeochemical cycling (Liesack et al., 2000). The small molecular weight organic and inorganic carbon compounds not exploited by other microbial competitors are then processed by methanogenic archaea to produce CH_4_ (Watanabe et al., 2007). Before its release to the atmosphere from paddy soils, the majority of CH_4_ is assimilated by methanotrophic bacteria. At oxic-anoxic interfaces in paddy soil, methanotrophic bacteria are able to oxidize up to 90% of total CH_4_ production (Bosse and Frenzel, 1997).

Due to ecological importance, paddy methanogenic archaea have been investigated extensively (Lu and Conrad, 2005; Angel et al., 2012; Bridgham et al., 2013). In particular, their responses and feedbacks to climate change are well documented. The consensus is that an elevated atmospheric CO_2_ will stimulate the growth of paddy methanogenic archaea, leading to more paddy CH_4_ emission (Liu G.C. et al., 2012). An elevated temperature can also increase paddy CH_4_ production (Peng et al., 2008). Recently, it has been found that elevated ground-level O_3_ negatively influences paddy methanogenic archaeal community (Feng et al., 2013). These studies set the foundation for our understandings about paddy methanogenic archaea. However, the foci of these investigations were mainly at the field-scale. The information about their geographical distribution at large scale, on the other hand, is quite scarce. Besides, little is known about the factors that drive paddy methanogenic archaea distribution (Ramakrishnan et al., 2001; Wang et al., 2010). Such knowledge can be useful in understanding their ecology.

Although no large-scale investigation has been conducted, several extant reports consistently have given the implications of the possible geographical distribution of paddy methanogenic archaea. By comparing the results from several field-scale investigations, it has been concluded that paddy methanogenic archaeal community composition could vary along geographic distance: the percentage of Methanocellaceae (Rice cluster I) in paddy methanogenic archaeal community decreases along latitude: 32.0% in Hainan Island, China (19.1°N) (Conrad et al., 2009a), 13.0% in Yangzhou, China (32.58°N) and 7.0% in Chikugo, Fukuoka, Japan (33.2°N) (Watanabe et al., 2009). Yan et al. (2003) estimated CH_4_ emission from rice fields in the East, Southeast and South Asian, finding that there is a strong geographical distribution of paddy CH_4_ emission, which is highly correlated with soil organic carbon (SOC) content. With the above phenomena, we hypothesize that there is a geographical distribution in paddy methanogenic archaeal community, and their distribution is related to spatial distance and environmental variables. To better understand the large-scale information of paddy methanogenic archaeal community it is worthwhile to explore the patterns more specifically.

**FIGURE 1:**
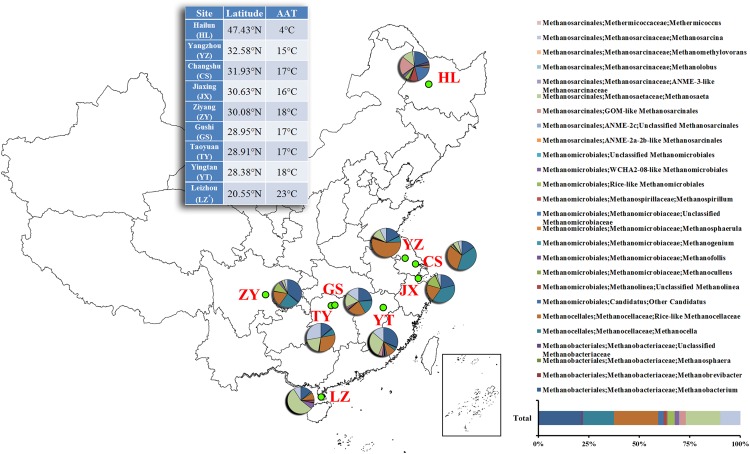
**Relative abundances of the dominant methanogenic archaea in each soil sample along latitudinal gradient in China.** The value of each genera percentage is the mean of triplicates. The inset of the 100% stacked column chart shows the percentages of all soils combined. The inset of the table shows the geographic information about each sampling site. *The abbreviation of each site is presented in parenthesis. AAT, annual average temperature.

China has nearly 34 million ha of paddy field and produces the highest annual rice yield among all countries. China also covers a wide geographic area, with a large temperature gradient zone from north to south. Such an unique characteristics allows a huge spatial and temporal variation in the content of SOC, along with their transformation (Yan et al., 2011). The geographic variation also leads to differences in the function and the diversity of associated soil microbes (Conant et al., 2011). Nine representative paddy soils (Leizhou, Yingtan, Taoyuan, Gushi, Ziyang, Jiangxi, Changshu, Yangzhou, and Hailun) along the latitudinal gradient (**Figure 1**) were collected. These sample locations were strategically selected from seven provinces that jointly hold roughly 60% of China paddy soil (Yan et al., 2003). Using pyrosequencing and real-time quantitative PCR (qPCR) assays, we have characterized the preliminary patterns of how paddy methanogenic archaeal communities are associated with their geographical distribution as well as the changing patterns of their abundance and diversity.

## Materials and Methods

### Site Selection and Soil Sampling

The geographic information about nine paddy sites sampled is listed in **Figure 1**. The temperature was annual average temperature, obtained from the website of Weather China^1^. These paddy sites are the major rice crop producing areas in China. Each of them has a history of more than 30 years of rice cultivation without substantial changes of agronomic practice. The rice of HL is inbred *Japonica* cultivar, while all others are inbred *Indica* cultivar. Soil samples were collected after the harvest of rice cultivation in 2012. In each site, soil sample had three replicates, which were 50 m away from each other. Each replicate of soil sample was collected at a depth of 0 to 10 cm at ten points along a zigzag line by auger boring. Aboveground plant materials, roots and stones were removed before homogenizing soil cores. Samples for molecular studies were maintained at -40°C until further use. Sub-samples for soil chemical assay were sieved and maintained at 4°C.

### Soil Chemical Properties Analyses

Soil pH was determined with AB15 pH meter (Accumet, Fisher Scientific). Inorganic N (NH_4_^+^-N and NO_3_^-^-N) were extracted by 2 M KCl and determined using a continuous flow analyzer (San^++^ System, Skalar, Netherlands) (Mulvaney, 1996). The contents of soil organic matter (SOM) and total N were determined by combustion (CNS-2000, LECO, St. Joseph, MI, USA) on dry soils ground with a ball mill (Retsch PM 200 Planetary Ball Mill, Haan, Germany) (Nelson and Sommers, 1982).

### Soil DNA Extraction

For each replicate of soil sample, genomic DNA was extracted from the same amount of moist soil (0.5 g) on the day after sampling using a FastDNA^®^ SPIN Kit for soil (MP Biomedicals, Santa Ana, CA, USA) according to the manufacturer’s instructions. The extracted soil DNA was dissolved in 50 μl of TE buffer, quantified by a spectrophotometer and stored at -20°C until further use.

### Real-Time Quantitative PCR

The copy numbers of methanogenic archaeal 16S rRNA genes [primer set 1106F (TTWAGTCAGGCAACGAGC)/1378R (TGTGCAAGGAGCAGGGAC)] of different samples were quantified by real-time qPCR following the protocols of Watanabe et al. (2007). Standard curves were obtained using 10-fold serial dilutions of the linear *Escherichia coli*-derived vector plasmid pMD18-T (TaKaRa) containing a cloned target gene, using 10^2^ to 10^8^ gene copies μl^-1^. The reactions were performed in C1000^TM^ Thermal Cycler equipped with CFX96^TM^ Real-Time system (Bio-Rad, USA). The 25-μl reaction mixture contained 12.5 μl of SYBR^®^ *Premix Ex Taq*^TM^ (TaKaRa), primer set (0.5 μM each), 200 ng BSA μl^-1^, 1.0 μl template containing approximately 2–9 ng DNA. Negative control was always run with water as the template instead of soil DNA extract. The qPCR program used for methanogenic archaea was: 94°C for 5 min, followed by 35 cycles of 94°C for 30 s, 55°C for 30 s and 72°C for 60 s, and extension and signal reading. The specificity of the amplification products was confirmed by melting curve analysis, and the expected sizes of the amplified fragments were checked in a 1.5% agarose gel. qPCR was performed in triplicate and amplification efficiencies of 97.4–104% were obtained with *R*^2^ values of 0.966–0.977.

### PCR and the Preparation of the Amplicon Libraries for Pyrosequencing

For each replicate of soil sample, the following primer set was used to amplify approximately 280 bp of methanogenic archaeal 16S rRNA gene fragments for sequencing on the 454 GS-FLX pyrosequencing platform: 1106F and 1378R (Feng et al., 2013). The oligonucleotide sequences included the 454 Life Science A or B sequencing adapters (19 bp) fused to the 7-bp bar-coded primer set as follows: Primer B (GCCTTGCCAGCCCGCTCAG) + barcode + forward primer; and Primer A (GCCTCCCTCGCGCCATCAG) + reversed primer. PCR was carried out in 50-μl reaction mixtures with the following components: 4 μl (initial 2.5 mM each) of deoxynucleoside triphosphates, 2 μl (initial 10 μM each) of forward and reverse primers, 2 U of *Taq* DNA polymerase with 0.4 μl (TaKaRa, Japan), and 1 μl of template containing approximately 50 ng of genomic community DNA as a template. Thirty-five cycles (95°C for 45 s, 56°C for 45 s, and 72°C for 60 s) were performed with a final extension at 72°C for 7 min. The purified bar-coded PCR products from all samples were normalized in equimolar amounts before pyrosequencing using Genome Sequencer FLX System platform (454 Life Science Branford, CT, USA). The sequences were deposited in NCBI database (accession no. SRP071098).

### Processing of the Pyrosequencing Data

The methanogenic archaeal 16S rRNA gene data were processed using the Quantitative Insights Into Microbial Ecology (QIIME) 1.7.0-dev pipeline^2^ (Caporaso et al., 2010) using default parameters unless otherwise noted. In brief, the sequences were denoised (Reeder and Knight, 2010) and binned into OTUs using a 97% identity threshold, and the most abundant sequence from each OTU was selected as a representative sequence for that OTU. Taxonomy was assigned to bacterial OTUs against a subset of the Silva 104 database. OTU representative sequences were aligned using PyNAST. A phylogenetic tree was then constructed using FastTree2 (Price et al., 2009) to the support phylogenetic diversity (PD) calculations.

A richness of phylotypes (Chao1) was calculated to compare community-level bacterial diversity at a single level of taxonomic resolution. We also estimated the PD using Faith’s index (Faith, 1992), which provides an integrated index of the phylogenetic breadth across taxonomic levels. We obtained total 74,921 methanogenic archaeal 16S rRNA sequences, and between 404 and 9,059 sequences per sample. Because an even depth of sampling is required for beta diversity calculations, we reduced the datasets to the lowest number available to correct for differences in survey effort between the samples. Namely, we calculated both diversity metrics using a randomly selected subset of 400 sequences per soil sample. This approach allows us to compare general diversity patterns among sites even though it is highly unlikely that we surveyed the full extent of diversity in each community (Shaw et al., 2008). The weighted pairwise UniFrac distances (Lozupone and Knight, 2005) were calculated for community comparisons using QIIME and were visualized using non-metric multidimensional scaling (NMDS) plots as implemented in PRIMER v6 (Clarke and Warwick, 2001).

### Statistical Analysis

Statistical procedures were calculated using the IBM Statistical Product and Service Solutions (SPSS) Statistics for windows (Version 13). The data were expressed as the means with standard deviation (SD), and the letters indicated significant differences between the results of the different samples. Mean separation was conducted based on Tukey’s multiple range test. Differences at *P* < 0.05 were considered statistically significant. Correlation between the abundances and the diversity of methanogenic archaeal community and environmental variables were quantified using analysis of variance (ANOVA). R software (Version 3.1.2) was utilized to estimate the correlations between environmental variables and methanogenic archaeal community composition by Mantel test and partial Mantel test (vegan package), to conduct Anosim, mrpp, and Adonis analyses (vegan package; Clarke and Warwick, 2001) and to build multivariate regression tree (MRT) to identify the most important abiotic factors for methanogenic archaeal community composition (mvpart package; De’Ath, 2002). MRT approach explains the variation of a single numeric response variable using explanatory variables that may be numeric and/or categorical. It is the natural extension of univariate regression trees, by summing the univariate impurity measured over the multivariate response. Mechanically, it grows a tree structure that partitions the data set into mutually exclusive groups. Starting with all the data represented by a single node at the top of the tree, the tree is grown by repeated binary splitting of the data. Each split is defined by a simple rule, usually based on a single explanatory variable, and forms two nodes. Splits are chosen to maximize the homogeneity of the resulting two nodes.

## Results

### Soil Chemical Properties of Nine Paddy Soils

The soil chemical properties of nine sampling sites are listed in **Table 1**. pH values ranges from 5.18 to 7.64, among which ZY and YZ had the highest values of 7.64 and 7.55 (*P* < 0.05), respectively. JX and TY had the highest NO_3_^-^-N values of 21.98 and 21.89 μg/g *d.w.s*, followed by ZY, YZ, CS, HL, GS, LZ, and YT. For NH_4_^+^ -N, Total N and SOM, GS had all highest values of 114.88 μg/g *d.w.s*, 0.26 and 4.80%, respectively (*P* < 0.05), and HL had the lowest values of 4.17 μg/g *d.w.s*, 0.11 and 2.19%, respectively (*P* < 0.05). Besides, HL had the highest C/N value of 11.99 (*P* < 0.05), followed by TY, LZ, CS, YZ, YT, GS, JX, and ZY.

**Table 1:** The soil chemical properties of nine sampling sites.

Site	pH	NO_3_^-^ -N (μg/g *d.w.s*)	NH_4_^+^ -N (μg/g *d.w.s*)	Total N (%)	SOM (%)	C/N
LZ	6.53 ± 0.09^b^	2.88 ± 0.04^g^	17.02 ± 0.26^e^	0.12 ± 0.00^g^	2.31 ± 0.01^g^	10.94 ± 0.13^bc^
YT	5.41 ± 0.01^f^	1.42 ± 0.20^h^	38.10 ± 3.96^d^	0.13 ± 0.00^f^	2.35 ± 0.01^g^	10.63 ± 0.11^d^
TY	5.18 ± 0.11^g^	21.89 ± 0.14^a^	97.76 ± 0.40^b^	0.23 ± 0.00^b^	4.31 ± 0.04^b^	11.01 ± 0.17^b^
GS	5.63 ± 0.04^e^	7.77 ± 0.04^f^	114.88 ± 0.41^a^	0.26 ± 0.00^a^	4.80 ± 0.04^a^	10.63 ± 0.15^d^
ZY	7.64 ± 0.14^a^	15.23 ± 0.08^b^	39.12 ± 0.50^d^	0.18 ± 0.00^d^	3.04 ± 0.05^e^	9.90 ± 0.12^e^
JX	6.16 ± 0.10^c^	21.98 ± 0.35^a^	65.89 ± 0.83^c^	0.19 ± 0.00^c^	3.25 ± 0.02^d^	10.13 ± 0.07^e^
CS	5.29 ± 0.02^fg^	13.98 ± 0.14^d^	2.88 ± 0.14^g^	0.15 ± 0.00^e^	2.84 ± 0.01^f^	10.92 ± 0.05^bc^
YZ	7.55 ± 0.04^a^	14.39 ± 0.19^c^	9.18 ± 0.42^f^	0.19 ± 0.00^c^	3.54 ± 0.02^c^	10.74 ± 0.21^cd^
HL	5.97 ± 0.06^d^	11.42 ± 0.21^e^	4.17 ± 0.30^g^	0.11 ± 0.00^h^	2.19 ± 0.01^h^	11.99 ± 0.20^a^

*The different letters in one column indicate significant difference.*

### Taxonomic Distribution of Methanogenic Archaeal Communities among Nine Paddy Soils

Pyrosequencing revealed that the paddy methanogenic archaeal community was dominated by two classes, Methanomicrobia (77.9%) and Methanobacteria (22.1%). With higher resolution, it was found that Methanocellales were most abundant (37.3%), followed by Methanosarcinales (30.2%), Methanobacteriales (22.1%) and Methanomicrobiales (10.4%), at the order level. At the family level, the dominant methanogenic archaea were found to be Methanocellaceae (Rice cluster I) (37.3%) and Methanobacteriaceae (22.1%), followed by aceticlastic groups Methanosaetaceae (17.2%) and Methanosarcinaceae (9.8%). At the genus level, the dominant methanogenic groups were Methanocella (Rice-like) (21.9%), Methanobacterium (21.1%), Methanosaeta (17.2%) and Methanosarcina (9.8%) (**Figure 1**; Supplementary Figure S1).

Each site has a different taxonomic distribution pattern (**Figure 1**). At the genus level, the percentage of *Methanosarcina* is significantly higher in TY (27.8 ± 1.6%) than in others (*P* < 0.05); the percentages of *Methanocella* are significantly higher in CS (39.6 ± 1.2%), JX (38.7 ± 3.3%), and ZY (23.3 ± 0.4%) (*P* < 0.05). Besides, LZ has the highest percentage of *Methanosaeta* (55.3 ± 17.4%) and YZ has the highest percentage of *Methanocella* (Rice-like) (55.1 ± 15.8%) (*P* < 0.05).

### The Abundance and Diversity Indices of Paddy Methanogenic Archaeal Communities against Soil Properties

We used qPCR to evaluate paddy methanogenic archaea abundances. YZ and ZY had the highest abundances (*P* < 0.05), with the copy numbers of 2.91 × 10^8^ and 2.00 × 10^8^ per *d.w.s*, respectively, followed by CS, TY, HL, JX, GS, YT, and LZ in order (**Table 2**). Paddy methanogenic archaeal diversity was evaluated by Chao1 and PD indices. Both indices had the similar changing patterns to each other among different paddy samples (**Table 2**). Briefly, HL and YT had the highest values of diversity indices (*P* < 0.05), followed by GS, CS, LZ, TY, ZY, and JX, and YZ had the lowest diversity values (*P* < 0.05).

**Table 2:** The abundances and diversity indices of methanogenic archaeal community in nine paddy sites.

	Abundance	Chao1	PD
**LZ**	6.13*(0.02)^e^	200.4 (17)^bc^	7.1 (0.3)^bc^
**YT**	7.35 (0.06)^d^	252.6 (17.5)^a^	7.8 (0.2)^a^
**TY**	7.92 (0.06)^c^	191.4 (11.6)^bc^	6.8 (0.6)^bc^
**GS**	7.77 (0.07)^c^	253.1 (12.6)^a^	7.4 (0.0)^ab^
**ZY**	8.30 (0.05)^ab^	189.9 (6.2)^bc^	6.5 (0.2)^c^
**JX**	7.80 (0.24)^c^	178.1 (56.9)^c^	6.5 (0.4)^c^
**CS**	8.00 (0.14)^bc^	226.2 (7.1)^ab^	6.9 (0.2)^bc^
**YZ**	8.46 (0.08)^a^	135.7 (6.3)^d^	5.7 (0.3)^d^
**HL**	7.90 (0.07)^c^	250.1 (23.9)^a^	7.9 (0.4)^a^

The different letters in one column indicate significant difference.

*it is the value of log_*10*_ of copy number.

We performed the correlations analysis between different environmental variables, the abundances of methanogenic archaeal community and their dominant groups, as well as their Chao1 and PD diversity indices (**Table 3**). It is shown that the abundance is significantly positively correlated with total N, SOM, NO_3_^-^-N, NH_4_^+^-N and atmospheric temperature (*P* < 0.05, Supplementary Figure S2), and negatively with C/N (*P* < 0.05). Combining the information of pyrosequencing and qPCR data, we analyzed the correlations of the absolute abundances of four dominant groups, Methanobacteriaceae, Methanocellaceae, Methanosaetaceae, and Methanosarcinaceae against soil properties (**Table 3**). They are generally correlated with C/N (*P* < 0.05, Supplementary Figure S3). Both diversity indices are dramatically negatively correlated with pH and NO_3_^-^-N, followed by atmospheric temperature (Supplementary Figure S4; *P* < 0.05). Besides, PD diversity index is significantly negatively correlated with Total N (*P* < 0.05) and positively with C/N (*P* < 0.05) (Supplementary Figure S5).

**Table 3:** The correlations between the soil properties and abundances of paddy methanogenic archaeal community, as well as diversity indices

		pH	NO_3_^-^-N	NH_4_^+^-N	Total N	SOM	C/N	Latitude	Temperature
**Total abundance**	*R*	0.040	0.483*	0.546**	0.673**	0.612**	-0.523**	-0.110	0.436*
	*P*	0.846	0.012	0.004	0.000	0.001	0.006	0.592	0.030
**Methanobacteriaceae**	*R*	0.314	-0.129	0.122	0.041	-0.065	-0.490*	0.252	-0.282
	*P*	0.118	0.529	0.553	0.844	0.760	0.011	0.215	0.171
**Methanocellaceae**	*R*	0.071	0.486*	0.251	0.557**	0.492*	-0.632**	-0.789**	0.871**
	*P*	0.731	0.012	0.217	0.003	0.011	0.001	0.000	0.000
**Methanosaetaceae**	*R*	-0.013	-0.547**	-0.477*	-0.481*	-0.376	0.662**	0.218	-0.342
	*P*	0.949	0.004	0.014	0.013	0.058	0.000	0.284	0.094
**Methanosarcinaceae**	*R*	-0.462*	0.053	0.344	0.133	0.232	0.579**	0.672**	-0.370
	*P*	0.017	0.798	0.085	0.518	0.253	0.002	0.000	0.068
**Chao1**	*R*	-0.615**	-0.475*	0.094	-0.289	-0.262	0.311	0.234	-0.391**
	*P*	0.002	0.022	0.671	0.182	0.226	0.148	0.276	0.000
**PD**	*R*	-0.552**	-0.531**	0.000	-0.426*	-0.371	0.499*	0.123	-0.381**
	*P*	0.006	0.009	1.000	0.043	0.081	0.015	0.542	0.000

*correlation is significant at the 0.05 level. **correlation is significant at the 0.01 level.

### Shift in the Composition of Methanogenic Archaeal Communities in Nine Paddy Sites

**FIGURE 2:**
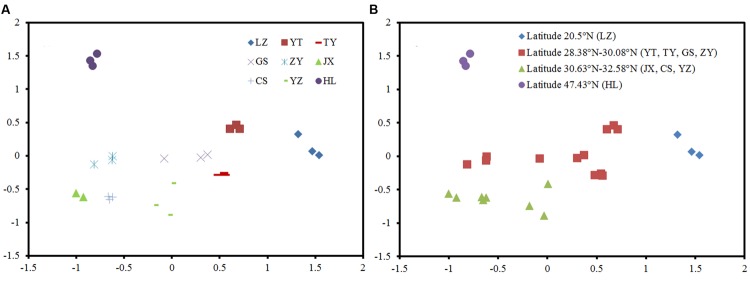
**Phylogenetic variability in the paddy methanogenic archaeal community composition among nine sites **(A)** and along latitude gradient **(B)**, indicated by a non-metric multi-dimensional scaling plot of the weighted pairwise UniFrac community distances between different soil samples based on 400 sequences per soil sample**.

The NMDS plots of the weighted pairwise UniFrac distance ordinations clearly demonstrated significant overall phylogenetic variability among paddy methanogenic archaeal communities in the nine sites we studied (**Figure 2A**). We found that the soil samples with close latitudes were grouped together (**Figure 2B**). Specifically, overall phylogenetic variability within paddy methanogenic archaeal communities at latitude ≤ 20.5°N (LZ), 28.38°N-28.95°N (YT, TY, and GS), 30.08°N-32.58°N (ZY, JX, CS, and YZ), and ≥47.43°N (HL) clustered separately from each other as shown in **Figure 2B**, which are confirmed by the analyses of Anosim, mrpp, and Adonis (Supplementary Table S1).

### Statistical Characterization of the Composition of Paddy Methanogenic Archaeal Communities against Soil Chemical Properties

Mantel test revealed the significant correlations between paddy methanogenic archaeal community composition based on the Bray–Curtis distance and the different environmental variables as well as spatial distance listed from the highest to the lowest correlation scores: temperature, C/N, spatial distance, NO_3_^-^-N, Total N and SOM (**Table 4**). These results are supported by canonical correspondence analysis (Supplementary Figure S6): latitude and C/N had the largest contributions to the first PC axes, and NO_3_^-^-N is the major contributor to the second PC axes. Partial mantel test further revealed that both spatial distance (*R* = 0.3469, *P* = 0.001) and soil chemical variables (*R* = 0.2847, *P* = 0.001) contribute to the variation of paddy methanogenic archaeal community composition, and spatial distance plays a more predominant role.

**Table 4:** The correlations between environmental variables and paddy methanogenic archaeal community composition (Bray–Curtis distance) determined by Mantel test.

Factor	*R*	*P*
Temperature	0.4768	0.001**
C/N	0.4183	0.001**
Spatial distance	0.3931	0.001**
NO_3_^-^-N	0.2887	0.001**
Total N	0.2631	0.002**
SOM	0.2092	0.01*
NH_4_^+^-N	0.04776	0.227
pH	0.0394	0.252

*indicates significant correlation. **indicates highly significant correlation.

**FIGURE 3:**
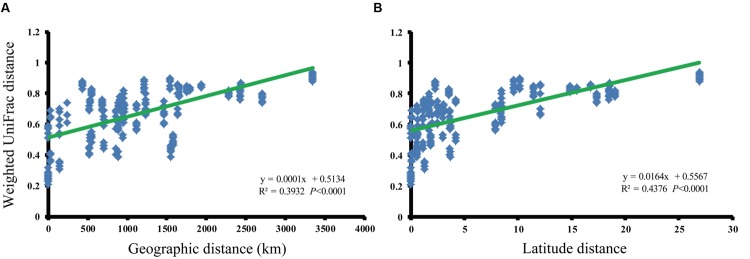
**The relationships between the phylogenetic distances of paddy methanogenic archaeal community and geographic **(A)** and latitude **(B)** distances as indicated by the weighted pairwise UniFrac differences in phylotype composition between sites**.

We analyzed the relationship between paddy methanogenic archaeal community composition (weighted pairwise UniFrac distances) and spatial distance (**Figure 3A**) as well as the latitude distances ranging from 0 to 27°N (**Figure 3B**), finding the obvious linear correlations (spatial distance: *y* = 0.0001x + 0.5134, *R*^2^ = 0.3932, *P* < 0.0001; latitude distance: *y* = 0.0164x + 0.5567, *R*^2^ = 0.4376, *P* < 0.0001). These phenomena indicate that paddy methanogenic archaeal community composition varied along spatial distance as well as latitude distance. Besides, a significant negative correlation between latitude and temperature is shown in Supplementary Figure S7.

**FIGURE 4:**
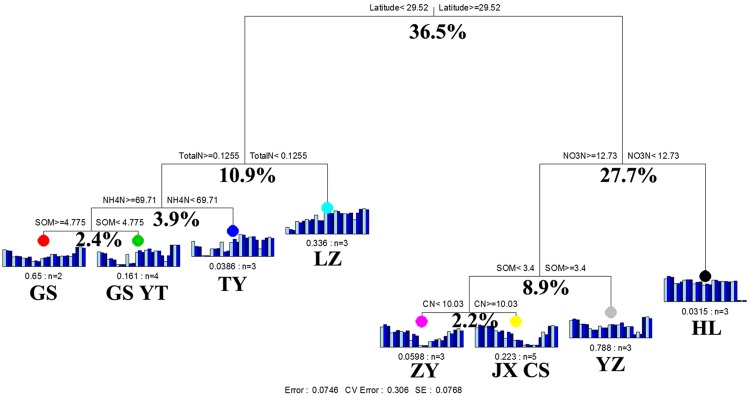
**Multivariate regression tree analysis of methanogenic archaeal communities in nine distant paddy sites**. Specific paddy sites and the number of samples included in the analysis are shown under bar plots. Numbers under the crosses of each split indicate percentages of variance explained by the split.

The results of MRT analysis are shown in **Figure 4**, which explains 92.5% of the detected variation among paddy methanogenic archaeal communities. Latitude produced the largest deterministic effects, contributing to 36.5% of the overall variations. Accordingly, the paddy methanogenic archaeal communities were clustered into two groups by latitude. The left group included GS, YT, TY, and LZ with their latitudes lower than 29.52°N, and the right group contained ZY, JX, CS, YZ, and HL (**Figure 4**) with latitudes higher than 29.52°N. This result partially echoed our Mantel test and partial Mental test findings of high correlation between spatial distance and paddy methanogenic archaeal community composition (Bray–Curtis distance, **Tables 4** and **5**). In the left group, the impacting factors of paddy methanogenic archaeal community were in the sequence of pH (10.9% of contribution), NO_3_^-^-N (3.9%) and SOM (2.4%). As a comparison, total N (8.9%) and pH (2.2%) are the two major contributors in the right group (**Figure 3**).

**Table 5:** Partial Mantel test of the effects of various factors on paddy methanogenic archaeal community composition.

Factor	*R*	*P*
Spatial distance	0.3469	0.001**
Environmental variables	0.2847	0.001**

**indicates highly significant correlation.

## Discussion

### Detailed Taxonomic Information on Paddy Methanogenic Communities

Generally, the methanogenic archaeal communities in nine paddy soils are dominated by the putative hydrogenotrophic phylotypes of Methanocellaceae (Rice cluster I) and Methanobacteriaceae, followed by the putative aceticlastic phylotypes of Methano saetaceae and Methanosarcinaceae (**Figure 1**; Supplementary Figure S1). The family Methanocellaceae (Rice cluster I) is the major producer of methane. Using RNA-based stable isotope probing analysis, Lu and Conrad (2005) found that Methanocellaceae (Rice cluster I) are the most active species for metabolizing rice root exudates and contribute greatly to paddy methane production. Methanobacteriaceae is also found to be the dominant methanogens in paddy soil (Kim et al., 2014). Acetate is most abundant in paddy soil, among low-molecular-weight organic acids (Penning and Conrad, 2006). With its concentration often exceeding 10 mM in anoxic paddy soil (Charmaine et al., 2010), acetate can promote the growth of aceticlastic methanogenic archaea. Therefore, it is not a total surprise when abundant aceticlastic methanogens, Methanosaetaceae and Methanosarcinaceae were observed in soil samples (**Figure 1**; Supplementary Figure S1).

This result is consistent with the findings of the members of paddy methanogenic archaeal community in South Korea and Japan by DGGE or T-RFLP, targeting 16S rRNA or *mcr*A genes (Kim et al., 2014; Liu et al., 2015). Using pyrosequencing, Hu et al. (2013) also found the same methanogenic archaeal constituents in paddy soils from South and North China. This consistency suggests that rice fields all over the world contain similar methanogenic archaeal constituents with respect to the major lineages (Ramakrishnan et al., 2001). However, it is noted that the proportions of each group are different, as well as their abundances (**Figure 1**; Supplementary Figure S1; **Table 2**). We believe the cause of this difference is not due to two rice cultivars (*Indica* and *Japonica*), as Ma et al. (2010) reported that the cultivar variety does not affect the abundance and composition of methanogen archaea. Instead, the difference is more likely to be a result of the different physico-chemical properties of niches (Conrad et al., 2008).

### Soil C and N Contents Determine the Abundance of Methanogenic Archaea

The total abundance of paddy methanogenic archaea varies among nine sampling sites (**Table 2**), but we found it is primarily correlated with soil C and N contents (**Table 3**). This finding is in agreement with results from the previous reports. Wang et al. (2010) and Watanabe et al. (2010) found that both the horizontal and the vertical distributions of the abundances of paddy methanogenic archaea are correlated with total soil C and N contents. Usually, higher SOC content readily enhances microbial substrates availability. This led Watanabe et al. (2010) speculate that such a correlation derives from the carbon substrates available to methanogenic archaea. For methanogenic archaea, they are the microbes responsible for the terminal C degradation in paddy soil. The high substrates derived from high SOC content can promote their growth. As evidence, climatic changes can influence the substrate availability, and hence affect the paddy methanogenic archaeal community (Liu G.C. et al., 2012; Feng et al., 2013). Besides, Liu D.Y. et al. (2012) found that DOC is the main factor predicting methanogenic archaea in wetland. And, we observed a positive correlation between the abundance of methanogenic archaea and SOM (**Table 3**). This phenomenon can well explain the strong positive correlation between paddy CH_4_ emission and SOM (Yan et al., 2003). Soil N contents can also influence paddy methanogenic archaea, due to two reasons. First, there is a positive correlation between SOM and soil total N (Huang et al., 2015). Second, the increase in N nutrient can promote crop growth, which increases root exudates and in turn stimulates the growth of methanogenic archaea; besides, the increased N nutrient can help soil microorganisms degrade SOM to release more electrons and carbon sources for methanogenic archaea to complete methanogenesis. Due to these reasons, our observation of positive correlations between the abundance and total N, NO_3_^-^-N and NH_4_^+^-N is not surprising (**Table 3**).

For dominant groups (i.e., Methanobacteriaceae, Methanocellaceae, Methanosaetaceae, and Methanosarcinaceae), the C/N ratio is generally the predominant factor that impacts their abundances (**Table 3**). Specifically, the abundances of hydrogenotrophic methanogens, Methanobacteriaceae (*R* = -0.490, *P* = 0.011) and Methanocellaceae (*R* = -0.632, *P* = 0.001) are negatively correlated with C/N, while aceticlastic methanogens, Methanosaetaceae (*R* = 0.662, *P* < 0.001) and Methanosarcinaceae (*R* = 0.579, *P* = 0.002) are positively correlated. This result is in line with the report of Wu et al. (2009) that the application of N fertilizer can increase hydrogenotrophic methanogens and decrease aceticlastic methanogens at the same time. Wu et al. (2009) offered the explanation that hydrogenotrophic methanogens (e.g., Methanocellaceae) inhabit rice roots where the competition for N element is intense between plant and microbes. An increase in N content benefits the growth of them all. We also found a positive correlation between Methanocellaceae abundance and NO_3_^-^-N or total N content (**Table 3**). The increase in hydrogenotrophic methanogens could outcompete aceticlastic methanogens for niche. Hence, a negative correlation between Methanosaetaceae abundance and N elements is observed (**Table 3**).

### pH Determines the Alpha Diversity of Methanogenic Archaeal Community

pH is a key variable in the soil environment, and differences in soil pH can arise from many factors (Lauber et al., 2008). Thus, soil pH naturally becomes a good predictor of soil microbial community (Fierer and Jackson, 2006). The importance of soil pH as a control on soil microbial community has frequently been demonstrated (Baker et al., 2009; Jesus et al., 2009; Jones et al., 2009; Lauber et al., 2009). Euyarchaeota community in paddy soil (Hu et al., 2013) and bacterial diversity in wetland (Hartman et al., 2008) are also found to be affected by soil pH. However, the influence of soil pH on paddy methanogenic archaeal community has hardly been studied before. In paddy soil, thermodynamically methanogenesis is the major sink of electrons or reducing equivalents (Kato et al., 2012). Low pH can activate trace elements in soil matrix and enhance their availability to soil microorganisms. Some of these trace elements, such as Fe can act as extracellular electron shuttle and promote the interspecies electron transfer from syntrophic bacteria to methanogenic archaea (Kato et al., 2012). For such reason, Kato et al. (2012) found that Fe amendment increases methanogenic archaeal abundance. In our study, we observed that pH is negatively correlated with the PD and Chao1 diversity indices of paddy methanogenic archaea, as well as the abundance of Methanosarcinaceae (**Table 3**).

### Both Spatial Distance and Soil Chemical Variables Play Important Roles in Driving Methanogenic Archaeal Community Composition

In this investigation, we found that both spatial distance and soil chemical variables, mainly focusing on soil C and N significantly contribute to the variation of paddy methanogenic archaeal community composition. Between these two factors, spatial distance plays a larger role (**Tables 4** and **5**; **Figure 3A**). Geographical distance has previously been described as one of important factors that determine microbial spatial distribution (Whitaker et al., 2003; Green et al., 2004). Such information is quite limited for paddy methanogenic archaea. Only a few studies have been reported in this area. For example, Ramakrishnan et al. (2001) found that the compositions of paddy methanogenic archaeal community in China, Philippines, and Italy are highly related with geography. Wang et al. (2010) found that the paddy methanogenic archaeal communities in high latitude (>42°N) in China are clustered by sampling sites. We speculated that the role of spatial distance is due to the different atmospheric temperatures of paddy soils sampled along the latitudinal gradient. Our analysis verified our speculation. First, paddy methanogenic archaeal communities can be grouped along latitudinal gradient (**Figure 2**). And, **Figure 3B** shows an obvious correlation between the biodistance of paddy methanogenic archaeal community composition and latitude distance. Secondly, the significant negative correlation between latitude and temperature is shown in Supplementary Figure S7. Besides, temperature appears to be the factor of the greatest influence on paddy methanogenic archaeal composition (**Table 4**). We believe the underlying mechanism is that the degradation process of SOM is sensitive to atmospheric temperature, which in turn influences the substrates of methanogenic archaea and governs their composition. Many reports demonstrated that temperature influences, by controlling both microbial metabolism and their substrate availability (Conant et al., 2011), the C and N mineralization functioning of soil general microbial community (Teklay et al., 2010), and/or their community composition (Feng and Simpson, 2009). More interestingly, Sun et al. (2013) conducted a large-scale reciprocal soil transplantation experiment, and examined microbial communities associated with straw decomposition in three initially identical soils placed in parallel in three regions of China. They found that atmospheric temperature is the pivotal factor determining the rate of straw decomposition and compositions of the associated soil microbial communities. Porazinska et al. (2012) reported that the richness of soil nematode is 300% more in the tropical than in the temperate rainforest. As we known, methanogenic archaea live at the terminal stage of C degradation of paddy soil. The variations in degradation processes of SOMs and the shifts in associated degrading microbes triggered by temperature would inevitably influence the composition of methanogenic archaeal community. We found Methanocellaceae to be positively correlated with temperature (**Table 3**). This is consistent with findings by others. For example, Fu et al. (2015) reported that Methanocellaceae (Rice cluster I), in response to elevated temperature in Zoige wetland, are more abundant at higher temperatures. Conrad et al. (2009b) also found that higher temperature can increase the dominance of Methanocellaceae (Rice cluster I).

Besides temperature, soil chemical variables (e.g., soil C and N contents) also have significant impacts on paddy methanogenic archaeal composition (**Tables 4** and **5**; **Figure 4**). This finding is similar to the report of Xiong et al. (2012). They also revealed the environmental variables as the second factor predicting the variation in soil bacterial community composition, following spatial distance. The variations in soil chemical variables could be due to different agricultural managements of sampling sites. The greatest determining factor according to MRT is latitude among all environmental factors, and paddy methanogenic archaeal community compositions can be segregated into two groups based on latitude of 29.52°N (**Figure 4**). This latitude is very close to the important geographical line of China (Qinling Mountains-Huaihe River line), which traditionally distinguishes the south (subtropical zone) and the north (warm temperate zone) China. For natural reasons, the north and the south adopt different agricultural managements, such as fertilization and water regimes, which can greatly influence soil physico-chemical properties and microbial diversities and functions. Consequently, paddy methanogenic archaeal composition is also influenced.

## Conclusion

We analyzed nine representative paddy soil samples in China and revealed that they harbor a phylogenetically diverse and quantitatively abundant methanogenic archaeal community, which is dominated by family Methano cellaceae, Methanobacteriaceae, Methanosaetaceae, and Methanosarcinaceae. Their abundance is influenced by soil C and N contents as well as alpha diversity by pH value. Both spatial distance and soil chemical variables, mainly about soil C and N, contribute to the variation of paddy methanogenic archaeal community composition, and the former could play a more predominant role. Because only several soil chemical parameters were focused, the relative importance of environmental variables on the variation in paddy methanogenic archaeal community composition at large scale might have been underestimated in this investigation. Meantime, spatial distance hosts a wide range of co-variables. Thus, a more robust and explicit investigation is needed to address the geographical distribution of paddy methanogenic archaea properly in future. As the ecologically important microbe, paddy methanogenic archaea have a critical role in ecosystem functioning and climate change. Thus the information on their biogeographic patterns would help us to get deeper insights into their ecology.

## Author Contributions

YF, XL, and ZJ designed the study. QZ and BW performed the experiments. QZ, YS, and YF analyzed the data. YF, LZ, YD, and QZ wrote the paper. All authors reviewed the manuscript.

## Conflict of Interest Statement

The authors declare that the research was conducted in the absence of any commercial or financial relationships that could be construed as a potential conflict of interest.

## Footnotes

1 http://www.weather.com.cn/

2 http://www.qiime.org
