# Supplementary material for: Geographical Distribution of Methanogenic Archaea in Nine Representative Paddy Soils in China
Source: Front Microbiol. 2016 Sep 13;7:1447. doi: 10.3389/fmicb.2016.01447 (PMC5020086; doi:10.3389/fmicb.2016.01447)
Supplement: Supplementary file 1 [file Data_Sheet_1.DOCX]

Supplementary Material

Distinct geographic patterns of abundance, diversity and the community composition of paddy methanogenic archaea in China

**List of authors:** Qianhui Zu, Linghao Zhong, Ye Deng, Yu Shi, Baozhan Wang, Zhongjun Jia, Xiangui Lin, Youzhi Feng^*^

***Correspondence:** Youzhi Feng: yzfeng@issas.ac.cn

**1. SI for Taxonomic distribution of methanogenic archaeal communities among nine paddy soils**


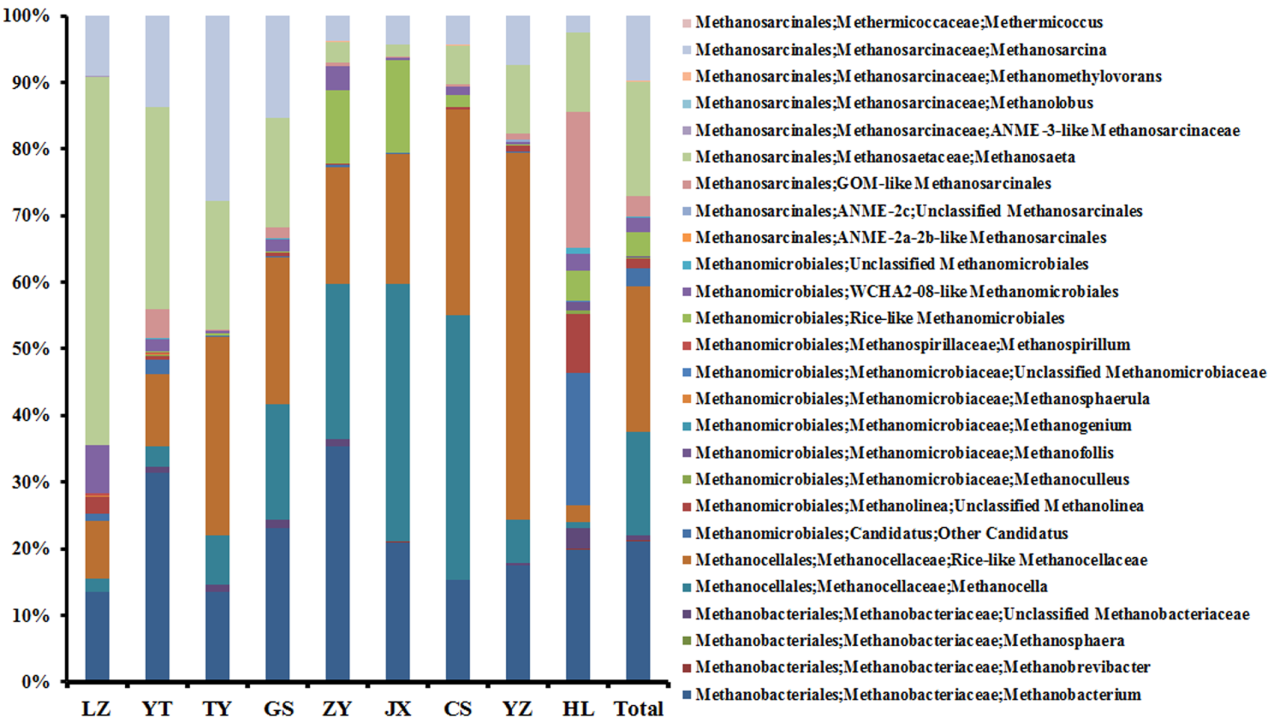


Figure S1. A 100% stacked column chart of the relative abundances of the dominant methanogenic archaea in each soil sample and all soils combined. The value of each phylum percentage is the mean of triplicates.

**2. SI for the correlation between methanogenic archaeal abundance and SOM as well as total N**

**
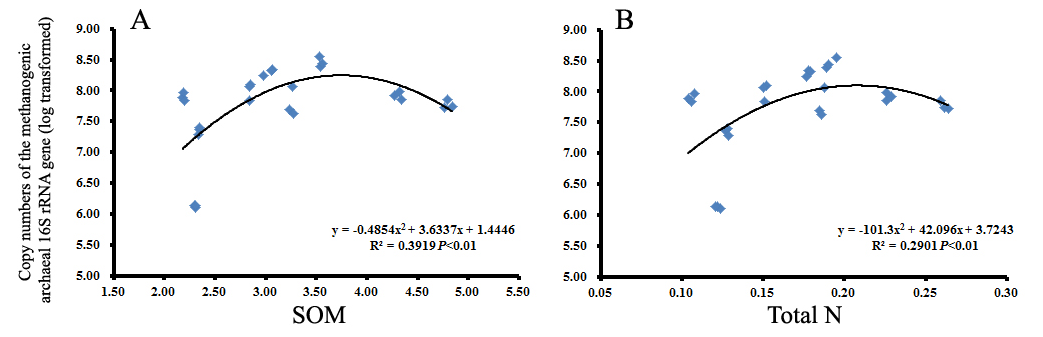
**

Figure S2. Copy numbers of the methanogenic archaeal 16S rRNA gene (log transformed) in relation to (A) SOM and (B) Total N.

**3. SI for the correlation between C/N and copy numbers of 16S rRNA gene of Methanobacteriaceae, Methanocellaceae, Methanosaetaceae and Methanosarcinaceae**


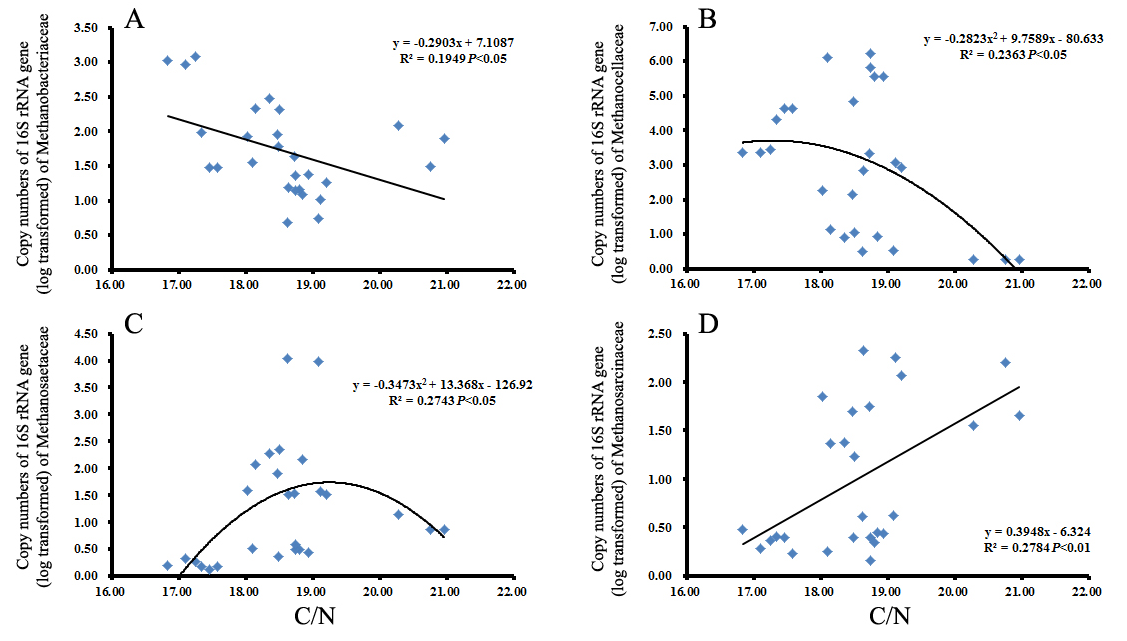


Figure S3. Copy numbers of 16S rRNA gene (log transformed) of (A) Methanobacteriaceae, (B) Methanocellaceae, (C) Methanosaetaceae and (D) Methanosarcinaceae in relation to C/N

**4. SI for the correlation between soil pH, NO_3_^-^-N and PD index as well as Chao1 index**


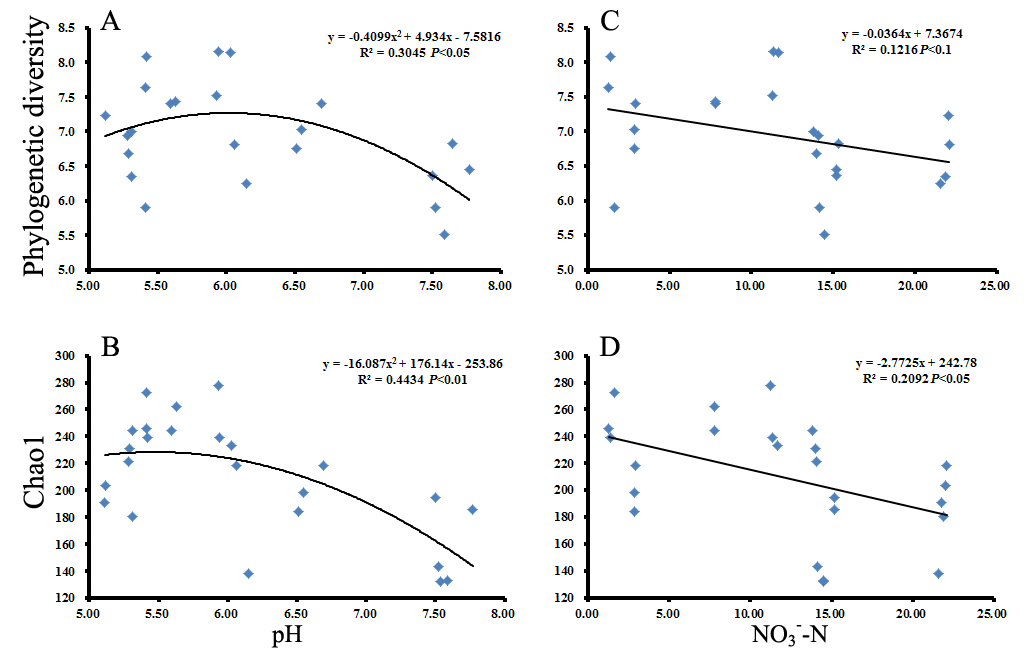


Figure S4. Soil pH and NO_3_^-^-N values in relation to (A and C) PD and (B and D) Chao1

**5. SI for the correlation between total N, C/N and PD index**

**
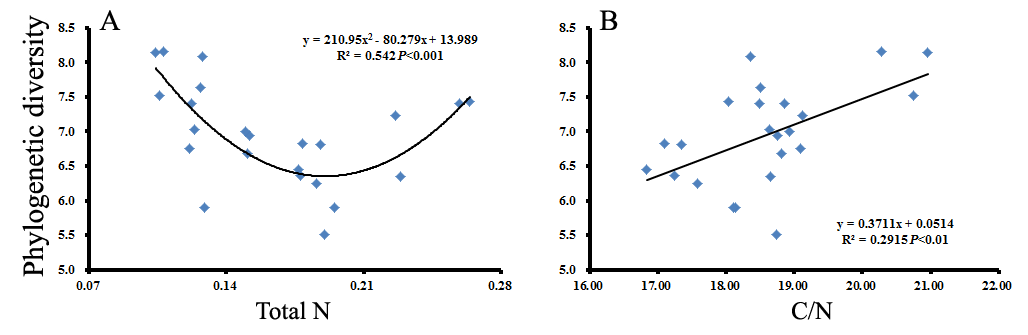
**

Figure S5. PD in relation to (A) total N and (B) C/N

**6. SI for the Anosim, mrpp and Adonis analyses of shifts in composition of methanogenic archaea**

Table S1. The Anosim, mrpp and Adonis analyses of shifts in composition of methanogenic archaea among latitude ≤ 20.5°N (LZ), 28.38°N-28.95°N (YT, TY and GS), 30.08°N-32.58°N (ZY, JX, CS and YZ), and ≥47.43°N (HL)

| Anosim | 1 | 2 | 3 | 4 |
| --- | --- | --- | --- | --- |
| 1 | - | - | - | - |
| 2 | 0.009 | - | - | - |
| 3 | 0.006 | 0.001 | - | - |
| 4 | 0.006 | 0.002 | 0.005 | - |
|  |  |  |  |  |
| mrpp | 1 | 2 | 3 | 4 |
| 1 | - | - | - | - |
| 2 | 0.005 | - | - | - |
| 3 | 0.003 | 0.001 | - | - |
| 4 | 0.003 | 0.004 | 0.004 | - |
|  |  |  |  |  |
| Adonis | 1 | 2 | 3 | 4 |
| 1 | - | - | - | - |
| 2 | 0.001 | - | - | - |
| 3 | 0.005 | 0.001 | - | - |
| 4 | 0.001 | 0.004 | 0.002 | - |

**7. SI for statistic characterization of methanogenic archaeal community compositions of nine paddy soils in China**


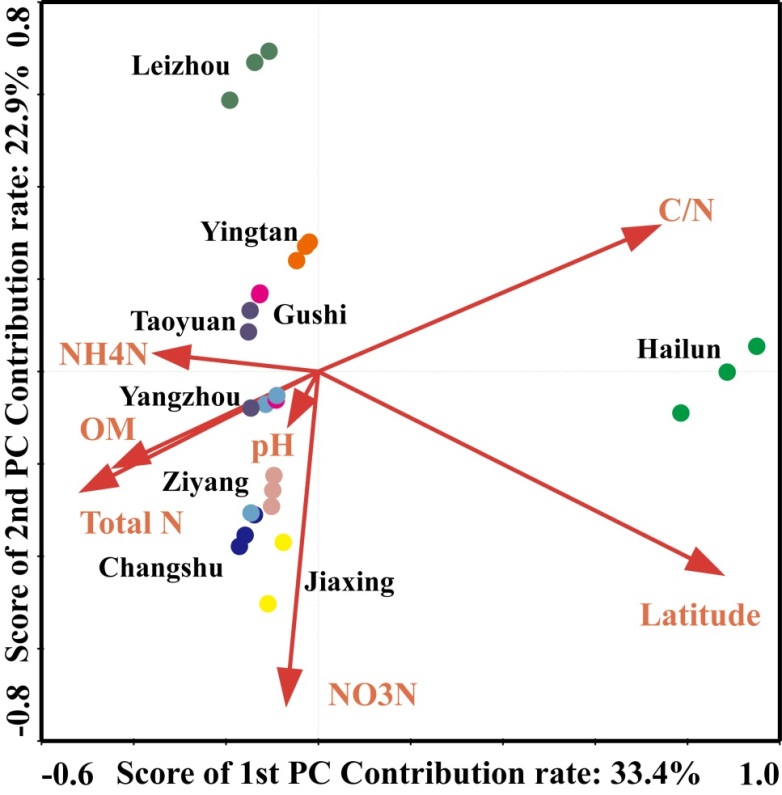


Figure S6. Canonical correspondence analysis relating paddy methanogenic archaeal community compositions with environmental variables in nine distant paddy soils. Axes 1 and 2 explain 33.4% and 22.9% of the total variance, respectively.

**8. SI for the negative correlation between latitudes and annual mean temperatures of nine paddy sites**


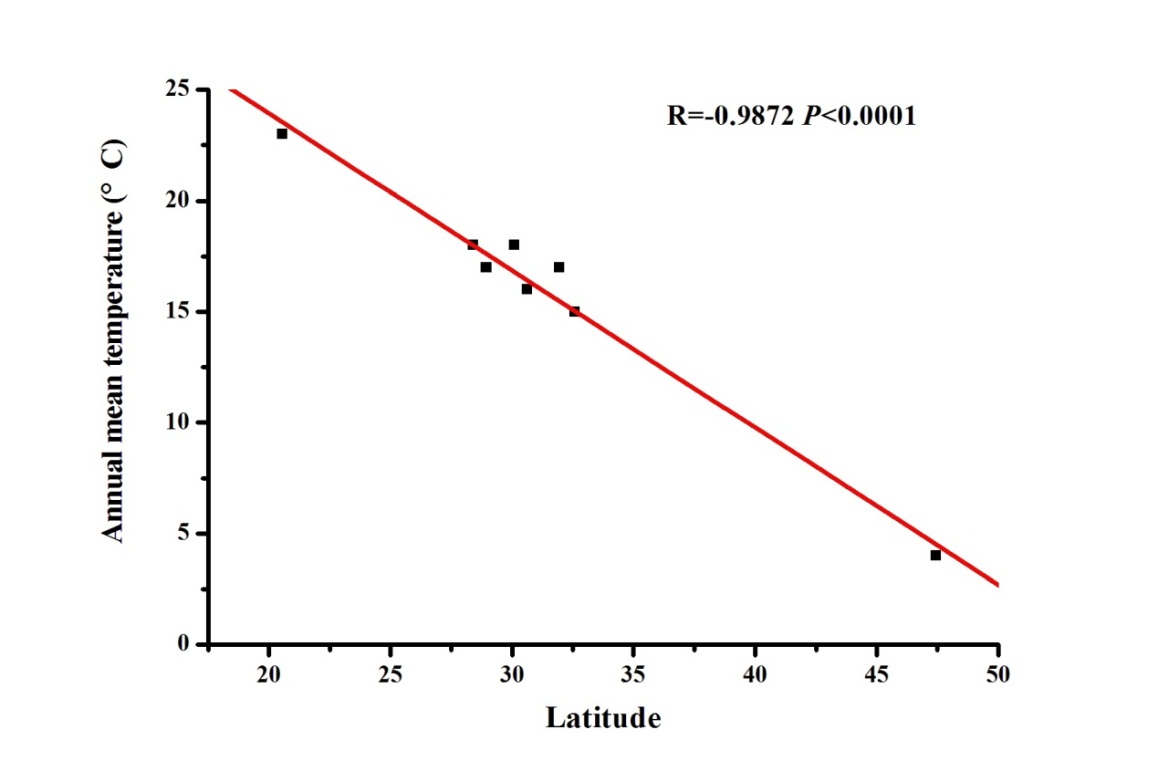


Figure S7. The correlation between latitudes and annual mean temperatures of nine paddy sites
